# Alterations in the hub structure of whole‐brain functional networks in patients with drug‐naïve schizophrenia: Insights from electroencephalography‐based research

**DOI:** 10.1002/pcn5.164

**Published:** 2024-01-10

**Authors:** Tomoaki Ishibashi, Sou Nobukawa, Mayuna Tobe, Mitsuru Kikuchi, Tetsuya Takahashi

**Affiliations:** ^1^ Department of Neuropsychiatry University of Fukui Fukui Japan; ^2^ Department of Computer Science Chiba Institute of Technology Chiba Japan; ^3^ Graduate School of Information and Computer Science Chiba Institute of Technology Chiba Japan; ^4^ Research Center for Mathematical Engineering Chiba Institute of Technology Chiba Japan; ^5^ Department of Preventive Intervention for Psychiatric Disorders, National Institute of Mental Health National Center of Neurology and Psychiatry Tokyo Japan; ^6^ Department of Psychiatry & Behavioral Science Kanazawa University Ishikawa Japan; ^7^ Research Center for Child Mental Development Kanazawa University Ishikawa Japan; ^8^ Uozu Shinkei Sanatorium Uozu Japan

**Keywords:** antipsychotic medication, betweenness centrality, electroencephalography, graph theory, schizophrenia

## Abstract

**Aim:**

This study aimed to identify atypical hubs in the whole‐brain networks of patients with schizophrenia (SZ) and examine the effects of antipsychotic medications, using electroencephalography (EEG) data.

**Methods:**

We estimated the functional connectivity across all electrodes by applying the phase lag index to the EEG signals of 21 drug‐naïve patients with SZ and 31 age‐matched healthy controls. Betweenness centrality (BC), a measure of hub status, was calculated for each electrode and frequency band. Data from 14 patients were re‐evaluated after initiating treatment with antipsychotic medications.

**Results:**

BC values decreased significantly at the Fz site in the beta band, decreased significantly at Pz in the gamma band, and increased significantly at O1 in the gamma band among patients with SZ. These changes persisted after antipsychotic treatment and were unrelated to clinical symptoms.

**Conclusion:**

The abnormal hub topology we observed, especially in the high‐frequency band, may reflect the pathophysiology of SZ, and this study highlights the utility of BC analysis of EEG data for detecting alterations in the whole‐brain networks of patients with SZ.

## Graphical abstract

This study investigated the presence of atypical hubs in the whole‐brain network of schizophrenia (SZ) patients using electroencephalography (EEG) data and examined the effects of antipsychotic medications. The results revealed significant changes in hub status, particularly in high‐frequency bands, among patients with SZ, which persisted after antipsychotic treatment. These findings suggest that abnormal hub topology in the whole‐brain network may reflect the pathophysiology of SZ, highlighting the utility of EEG‐based betweenness centrality analysis for detecting altered brain networks of SZ.

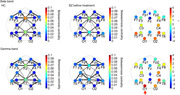

## INTRODUCTION

Schizophrenia (SZ) manifests with hallucinations, delusions, disorganized speech and behavior, as well as negative symptoms involving withdrawal, decreased motivation, and cognitive deficits.^1^, ^2^ Cognitive deficits are a major field of interest in studies on SZ and are attributed to intense or continued psychiatric symptoms.^3^ However, these deficits are understudied and are not included in the diagnostic criteria for SZ.^4^ Nevertheless, diagnostic tools for neurocognitive evaluation and years of observational studies have revealed that cognitive deficits occur in the early phase of SZ and are directly associated with poor functional outcomes.^5^, ^6^ Conventional treatments, including pharmacotherapy and psychosocial treatment, have not significantly improved cognitive deficits.^7^, ^8^ Although aberrant neural networks reportedly contribute to cognitive deficits, the exact underlying mechanisms remain unclear.

The notion of brain dysfunction in SZ originated from the term “schizophrenia” (schizo: divided, phrenia: mental) coined by Bleuler. The disconnection hypothesis was proposed as an expression of the pathophysiology of SZ, and it has been validated by several clinical studies.^9^, ^10^ Recently, studies in SZ have attempted to elucidate the associated abnormalities in whole‐brain functional networks,^11^ in addition to conventional studies that have investigated the pathophysiology of SZ from a microscopic perspective by focusing on the dopamine hypothesis or glutamate hypothesis.^12^, ^13^ Functional magnetic resonance imaging (fMRI) is a widely used neuroimaging modality for investigating the functional network. However, fMRI signals are an indirect measure of neural activity and have a limited temporal resolution. Contrarily, electroencephalography (EEG) is a convenient and noninvasive technique that directly detects the electrical fields that the cortex generates with excellent temporal resolution. Therefore, EEG has been effectively used to capture instantaneous functional connectivity and has long been used to detect aberrant neural networks generated in patients with SZ.

EEG dynamics at different temporal scales and frequency bands (such as the alpha, beta, gamma, and theta bands) are associated with different memory, cognitive, and perceptual functions.^14^ Recently, graph theory‐based approaches have been applied to EEG data analysis, revealing the hub structure of whole‐brain functional networks that plays an essential role in cognitive functions. SZ is characterized by abnormalities in the interactions between regions of the whole brain, therefore changes in the topological characteristics of the whole brain are more important to investigate than changes in the connectivity of individual regions. From this perspective, Takahashi et al. have used the phase lag index (PLI) to clarify anomalies in the SZ brain network in each EEG band.^15^

Human cognitive functions are not simply based on sensory receptor signals (bottom‐up information) but are also modified by memory, experience, and emotional brain signals (top‐down information). This fact is increasingly recognized in neuroscience.^16^, ^17^, ^18^ The gamma‐band oscillation has been associated with bottom‐up processes, whereas top‐down processes are likely mediated by beta‐band oscillation.^19^, ^20^ It has long been noted that an imbalance between top‐down and bottom‐up information can cause hallucinations and illusions.^21^ Moreover, several studies on SZ have shown that such imbalances are related to the background of psychiatric symptoms.^22^, ^23^, ^24^, ^25^ Changes in the whole‐brain network that underlie various symptoms of SZ may comprehensively explain the pathology of the disease. A more detailed analysis of these differences from healthy controls—or from patients with other psychiatric disorders—may provide objective biomarkers and information that can be used to develop efficient methods of therapeutic intervention. Previously, we identified abnormal functional connectivity in patients with SZ using graph theoretical analysis of EEG data.^15^ Specifically, the PLI, which captures the true synchronization of paired EEG signals, was used to discover diminished functional connectivity in the beta band across frontal regions and in the gamma band throughout the brain among patients with SZ in comparison to control participants. However, PLI evaluates the strength of the association between the two electrodes and is limited in capturing whole‐brain network features. Here, we identified atypical hubs of the neural network in patients with SZ and evaluated their responses to antipsychotic medications. Accordingly, betweenness centrality (BC; a measure of hub status), which is more suitable than the PLI for evaluating the importance of each node in terms of the entire network from the viewpoint of minimum paths, was used in post‐PLI analysis.

## MATERIAL AND METHODS

This research involves a secondary analysis of data acquired in a prior study,^15^ in which we revealed beta and gamma band‐specific reductions of PLI values in SZ in comparison with HC. To avoid duplication, the results presented in previous study, including the PLI figures, are not included here, therefore the reader is referred to the previous study for details of the PLI analysis.

### Participants

The clinical group comprised 21 individuals diagnosed with SZ who were recruited from the outpatient department of Kanazawa University Hospital in Ishikawa, Japan. Diagnosis of SZ was made in accordance with the criteria outlined in the *Diagnostic and Statistical Manual*, 4th edition (DSM‐IV). Patients with concurrent neuropsychiatric conditions or those under ongoing medication were excluded. Subsequently, seven patients from the SZ group were excluded during the second EEG session, 2–6 weeks following the initiation of antipsychotic treatment, primarily due to their refusal to continue participation or to worsening of psychotic symptoms. In contrast, the healthy control (HC) group consisted of 31 individuals with no reported history of neuropsychiatric disorders. These individuals were recruited from both the staff of Kanazawa University Hospital and their family members. While SZ and HC groups were matched based on age and gender, their educational backgrounds varied. The Brief Psychiatric Rating Scale (BPRS) was employed to assess the symptoms of each patient on the day of the EEG recording, and this assessment was performed by the same clinician both before and after treatment. The demographic features of each group are outlined in Table 1. All participants who agreed to participate were made aware of the research and provided written informed consent. The Kanazawa University Ethics Committee approved the study and it adhered to the Declaration of Helsinki.

**Table 1:** Demographic characteristics of participants.

	Healthy controls (*n* = 31)	Patients with schizophrenia before treatment (*n* = 21)	Patients with schizophrenia after treatment (*n* = 14)
Women/men	15/16	10/11	9/5
Age, years	27.9 (8.2)	28.1 (10.1)	29.5 (9.6)
Education, years	15.9 (2.0)	14.1 (1.9)	13.7 (1.5)
Duration of illness, months	Not Available	24.2 (36.2)	23.6 (42.0)
BPRS before treatment	Not Available	52.6 (13.2)	56.2 (13.2)^a^
BPRS after treatment	Not Available	Not Available	43.2 (14.6)^a^
Risperidone equivalent dose of antipsychotics	Not Available	Not Available	3.2 (1.9)

*Note*: Values represent mean (SD).

Abbreviations: BPRS, Brief Psychiatric Rating Scale; EEG, electroencephalography; NA, SD, standard deviation.

^a^ Paired‐*t*‐test between BPRS before and after treatment shows a significant difference (*t*[13] = 2.989, P = 0.010).

### EEG recordings

The EEG data were recorded using 16 electrodes based on the International 10‐20 system, which included Fp1, Fp2, F3, F4, Fz, F7, F8, C3, C4, P3, P4, Pz, T5, T6, O1, and O2. The reference electrodes were situated on the connected earlobes, and an additional electrooculogram was employed to monitor eye movements during EEG recordings. The impedance of each electrode was carefully maintained below 5 kΩ. The EEG data were sampled at 200 Hz and bandpass filtered at 1.5–60 Hz. An 18‐channel system (EEG‐44189; Nihon Kohden) was utilized for storing the EEG data offline. Participants were instructed to recline in a soundproof, light‐controlled, and electrically shielded recording room with their eyes closed. EEG signals were meticulously inspected for the identification of artifacts such as muscle activity, blinks, and eye movements. Continuous epochs of 600 s free from artifacts were extracted from each participant's data. Subsequently, bandpass filtering was carried out for each epoch to separate the conventional frequency bands, including delta (2–4 Hz), theta (4–8 Hz), alpha (8–13 Hz), beta (13–30 Hz), and gamma (30–60 Hz). To eliminate the line noise at 60 Hz introduced by the notch filter, a bandpass was applied. A 4000th‐order bandpass finite impulse response (FIR) filter with a linear phase was designed, utilizing the Hamming window. For PLI analysis, the use of excessively long epochs hinders the identification of disease‐specific changes since the values decrease with increasing epoch length.^26^ Conversely, adopting short epochs may fail to capture behaviors characterized by slow frequency components. To address this, each epoch was subdivided into 5‐s intervals for the purpose of balance.^15^, ^27^ The initial and final epochs were excluded from subsequent connectivity analysis to enhance the stability of the EEG signals.

### PLI

Functional brain networks were formed utilizing the PLI—a metric that attenuates artifactual interactions—to estimate functional connectivity between electrodes.^28^ For each epoch, the instantaneous phase at each time point of the band‐passed‐filtered waveform was calculated with the Hilbert transform. Subsequently, the phase difference between electrodes, $\Delta \varphi (t_{k})$ [rad] (*k* = 1,2,3, …, *T*, where *T* is the number of time points in an epoch), was calculated. The PLI between the pair‐wise electrodes was obtained with the following equation in each epoch:

$$\text{PLI}=\left|\frac{1}{T}\sum_{k=1}^{T}\text{sign}\Delta \varphi (t_{k})\right|$$

PLI is a measure of the concordance rate that represents the degree of phase difference, and its values range from 0 to 1, where 0 means no coupling or zero‐lag coupling, and 1 means perfect phase coupling. In particular, with low PLI values, $\Delta \varphi (t_{k})$ distributes homogeneously in the phase space or at around zero and π [rad], while with high PLI values, $\Delta \varphi (t_{k})$ distributes at around a specific phase difference that is not zero or π [rad].

### BC

For both groups of participants, we calculated BC at each electrode and frequency band. BC is an indicator that can be used to identify focal nodes in a network^29^ and is superior to other indicators in that it is able to assess not only neighborhood connectivity relationships, but also broader network trends, highlighting already reported SZ network abnormalities.^15^ Mathematically, the definition of BC is the ratio of the number of shortest paths crossing a node to the total number of shortest paths within the network. The BC estimation of functional connectivity requires the path length between electrodes; a stronger degree of coupling synchronization corresponds to a shorter distance between nodes. In other words, a higher PLI value results in a shorter path length of functional connectivity. Here, the length of a path between a pair of electrodes was defined as the inverse of the PLI value. Nodes with high BC values play an important role as hubs and correspond to bridge nodes in the network.

The BC of node i ($b_{i}$) is defined as follows:

$$b_{i}=\frac{1}{\left(n-1\right)\left(n-2\right)}\sum_{h,j\in N,h\neq j,h\neq i,j\neq i}\frac{\rho_{\mathrm{hj}}(i)}{\rho_{\mathrm{hj}}}$$

where $\rho_{\mathrm{hj}}$ represents the number of shortest paths from node h to node j, $\rho_{\mathrm{hj}}(i)$ represents the number of shortest paths passing through node i, N is the set of all nodes in the network, and n is the number of nodes. The value of $b_{i}$ is normalized to 0 and 1 via division by (n−1)(n−2). We used MATLAB's Brain Connectivity Toolbox ^30^ to calculate BC. In several studies, BC has been calculated against the minimum spanning tree (MST) of the functional connectivity to focus only on the main network backbone.^31^, ^32^, ^33^, ^34^ However, the pruning process of MST removes weak connections, which can affect the global pathways, therefore here we derived the BC from whole‐brain connections to comprehensively analyze functional connectivity.

### Statistical analysis

We conducted a repeated measures analysis of variance (ANOVA) to compare the pretreatment BC values between the HC and SZ, with group (HC vs. SZ before treatment) as the between‐participants factor and interaction of nodes (16 electrodes) as the within‐participants factor at each frequency band. First, the Greenhouse–Geisser adjustment was applied to the degrees of freedom. A two‐sided *α* of 0.05 was considered statistically significant to avoid type I errors. Then, we performed post hoc *t*‐tests controlled by the Benjamini–Hochberg false discovery rate (FDR) procedure^35^ for the BC values of all electrodes (HC vs. SZ before treatment) to identify the BC values with significant main and interaction effects. Following this, *t* values adjusted to *q* < 0.05 against the frequency bands with significant effects were used for further analysis. We employed a repeated measures ANOVA with treatment condition (SZ before treatment vs. SZ after treatment) as a within‐participant factor and interaction of nodes (16 electrodes) as a within‐participant factor at each frequency band.

The Greenhouse–Geisser adjustment was applied to compare the pre‐ and posttreatment BC values in the SZ group to the degrees of freedom, and a two‐sided *α* of 0.05 was considered statistically significant. Next, BC values with significant main (treatment) and interaction (treatment × node) effects were used for post hoc paired *t*‐tests controlled by the FDR procedure.^35^ Following this, *t* values adjusted to *q* < 0.05 against the frequency bands with significant main (treatment) and interaction (treatment × node) effects were used for further analysis. Finally, we also evaluated the relationship between changes in BPRS and improved BC values by calculating Pearson's correlation coefficient (R) between these factors. A significant correlation was shown by P<0.05.

We calculated Pearson's correlation coefficient (R) between these factors to evaluate the relationship between BC values and the severity of disease in SZ (as measured by BPRS scores), and between BC values and age. A significant correlation was indicated by P<0.05.

## RESULTS

**Figure 1:**
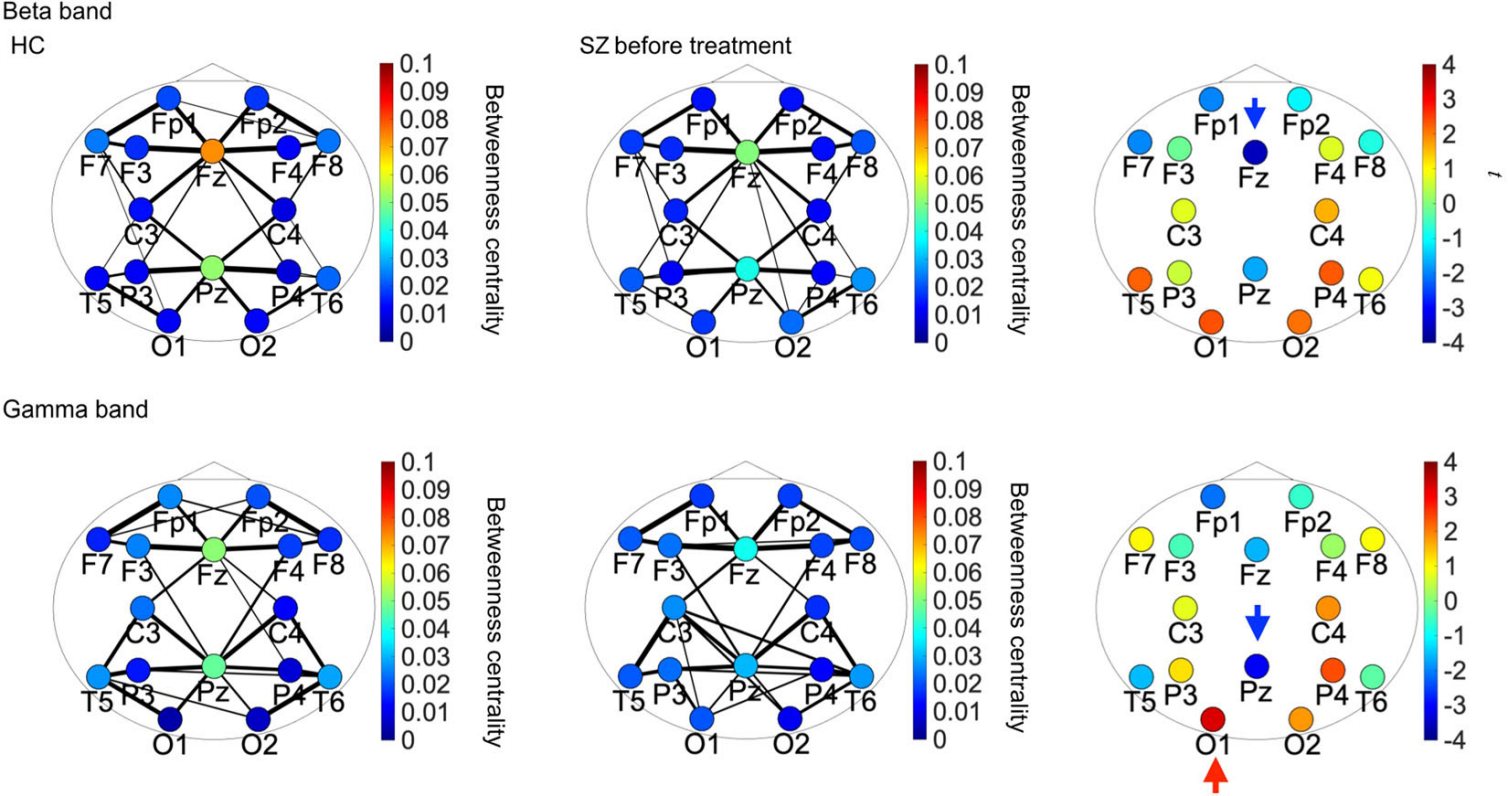
(a) Mean betweenness centrality (BC) values in the healthy control (HC) and pretreatment schizophrenia (SZ) groups in the beta and gamma bands. Phase lag index (PLI) values with strength in the top 20% at each frequency band are shown for each electrode pair. The width of the circles corresponds to the strength of the PLI value. In both groups (HC and SZ), the Fz and Pz electrodes show high BC values in the beta and gamma bands. (b) the *t* values of comparisons between the pretreatment SZ and HC groups in the beta and gamma bands ARE SHOWN. High (low) *t* values correspond to high (low) BC values in the pretreatment SZ group compared with those in the HC group. The results show a significant decrease in the BC values of Fz in the beta band (*q* < 0.05, blue arrow), a decrease in the BC values of Pz in the gamma band (*q* < 0.05, blue arrow), and an increase in the BC values of O1 in the gamma band (*q* < 0.05, red arrow).

**Figure 2:**
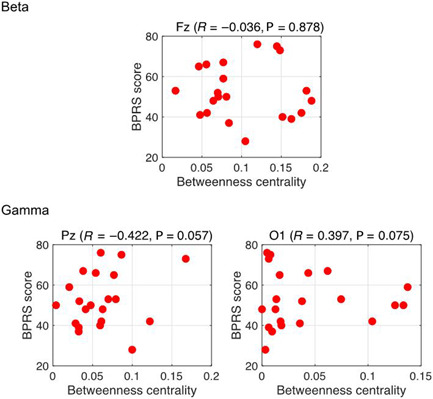
Scatter plots of pretreatment betweenness centrality values versus BPRS scores in the schizophrenia group, with Pearson's correlation coefficients (*R*). No significant correlations are observed. BPRS, Brief Psychiatric Rating Scale.

The connectivity indices obtained before calculating BC (i.e., the PLI) were validated in our previous study.^15^ We evaluated the between‐group differences in pretreatment BC values in the delta, theta, alpha, beta, and gamma bands (Table 2). We found a significant main effect of group in the gamma band and a significant interaction effect with node in the beta and gamma bands. To identify the BC values with significant main and interaction effects, we visualized mean pretreatment BC values in the HC and SZ groups in the beta and gamma bands (Figure 1a). We visualized the PLI values for each frequency band for each electrode pair with connectivity strength in the top 20%. The Fz and Pz electrodes showed high BC values in the beta and gamma bands in both groups (HC and pretreatment SZ). The results of the post hoc *t*‐test are shown in Figure 1b. The findings demonstrated a notable decrease in the BC value of Fz in the beta band (*q* < 0.05), a decrease in the BC value of Pz in the gamma band (*q* < 0.05), and an increase in the BC value of O1 in the gamma band (*q* < 0.05). We generated a scatter plot of symptom severity (BPRS scores) versus BC and calculated the Pearson's correlation coefficient (*R*) (Figure 2), observing no significant correlations. We used the same process to evaluate the relationship between BC and age, and again found no significant correlations. In the area of interest listed above (Fz in the beta band, Pz in the gamma band, and O1 in the gamma band), weak correlations of BC and age were seen only in HC gamma. Evaluating the relationship between these BC values and BPRS scores in the SZ group for O1 gave *ρ* = −0.45 and P = 0.11.

**Table 2:** Results of a repeated measures ANOVA for betweenness centrality differences between the healthy control and pretreatment schizophrenia groups in the delta, theta, alpha, beta, and gamma bands.

Band	*F*	P	*η* ^2^
*Delta*			
Group	1.567	0.217	0.3
Group × node	0.803	0.616^a^	0.016^b^
*Theta*			
Group	0.449	0.506	0.009
Group × node	0.841	0.581^a^	0.017^b^
*Alpha*			
Group	0.004	0.947	0
Group × node	0.616	0.692^a^	0.012^b^
*Beta*			
Group	2.083	0.155	0.04
**Group** × **node**	**4.538**	**<0.001** a	**0.083** b
*Gamma*			
**Group**	**4.358**	**0.042**	**0.08**
**Group** × **node**	**4.27**	**0.044** a	**0.079** b

*Note*: Significant main and interaction effects (P < 0.05) are in bold.

^a^ Corrected by false discovery rate (Benjamini–Hochberg procedure).

^b^ Corrected using the Greenhouse–Geisser method.

We also evaluated the differences in the SZ group's pre‐ and posttreatment BC values (Table 3). However, we found no significant main (treatment) or interaction (treatment × node) effect, nor any significant association between BPRS change and improved BC values.

**Table 3:** Pre‐ versus posttreatment differences in betweenness centrality within the schizophrenia group: Results of a repeated measures ANOVA in the delta, theta, alpha, beta, and gamma bands.

Band	*F*	P	*η* ^2^
*Delta*			
Before vs. after	0.007	0.935	0.001
Node	7.222	<0.001	0.357
Node × before vs. after	0.981	0.441^a^	0.07
*Theta*			
Before vs. after	0.523	0.482	0.039
Node	5.721	<0.001	0.306
Node × before vs. after	0.641	0.714^a^	0.047
*Alpha*			
Before vs. after	1.56	0.234	0.107
Node	13.449	<0.001	0.508
Node × before vs. after	1.34	0.265^a^	0.093
*Beta*			
Before vs. after	0.007	0.935	0.001
Node	11.558	<0.001	0.471
Node × before vs. after	0.45	0.843^a^	0.033
*Gamma*			
Before vs. after	0.098	0.759	0.008
Node	3.097	0.021	0.192
Node × before vs. after	0.979	0.44^a^	0.07

*Note*: No significant main (treatment) or interaction (treatment × node) effects WEre observed.

^a^ Corrected by false discovery rate (Benjamini–Hochberg procedure).

## DISCUSSION

We analyzed EEG data using PLI‐based BC values (a measure of hub status) to examine the changes in resting‐state functional connectivity in drug‐naïve patients with SZ. Our results revealed frequency‐ and region‐specific abnormalities in the hubs of neural networks, and the results were especially evident in the high‐frequency bands. The clinical symptoms of SZ (as measured by BPRS) were not correlated with BC values, and we observed no significant differences in BC values before and after medication.

Functional connectivity patterns in patients with SZ are reportedly atypical, but findings have been inconsistent. This may be due to differences in the methods of analysis used or patient backgrounds (including the effects of medication and disease symptoms).^36^ In particular, EEG studies have reported a significant impact of medication on network analysis results.^37^, ^38^ Additionally, Takahashi et al. showed that in patients with SZ, abnormal EEG signal complexity in the forebrain region (as confirmed by multiscale entropy analysis) was selectively normalized by antipsychotic treatment.^39^ Generally, these results show that functional connectivity is directly affected by medication, therefore analyzing data from drug‐naïve patients and evaluating the effects of medication on whole‐brain networks may provide useful insights into the pathophysiology of the disease.

Patients with SZ show significant changes in their brain network organization, as indicated by graph‐analytical measurements of global short communication paths, local organization, and small‐worldness.^40^ A growing number of studies, including both anatomical and neurophysiological approaches using diffusion tensor imaging (DTI), fMRI, and EEG, have focused on hub status and have identified atypical network patterns. The importance of abnormalities in brain hubs is established and such abnormalities comprise the background of many psychiatric disorders.^41^ Regarding anatomical alterations of white matter structure examined by DTI, connections between hub regions comprising the “rich club” were disproportionately affected in patients with SZ, a difference that was not ameliorated with medication.^42^ In an fMRI study, frontal hubs of patients with SZ showed a significant reduction in BC values.^43^ Cheng et al. also showed that changes in functional hubs are associated with SZ and that BC values can identify patients with SZ with a high level of accuracy.^44^ The decreased BC values in the frontal lobes observed in these studies are consistent with our results. Furthermore, Crossley et al.^41^ performed a meta‐analysis of 314 task‐based functional neuroimaging studies. They reported that patients with SZ showed abnormal hub structure and decreased hub function in specific brain regions. They indicated that these abnormalities increased compensatory activity in other brain regions.^45^ The established anatomical locations of under‐ and overactivations in SZ are consistent in crucial respects with our results.

In contrast to fMRI studies, relatively few EEG studies examine the hub structure of whole‐brain networks. Krukow et al. analyzed the EEG signals of first‐episode patients with SZ and demonstrated increased gamma band BC in posterior nodes and a correlation between these BC values and cognitive deficits.^46^ This is consistent with our findings of increased BC in O1. However, Krukow et al. found no differences in other frequency bands or nodes between patients with SZ and controls. In a comparison of neural network topologies between patients with first‐ and multi‐episode SZ,^47^ significant between‐group differences in maximal BC and tree hierarchy were observed in both the beta and the gamma bands. On the basis of these results, the authors showed that the duration of illness significantly affects the topology of resting‐state functional networks. Despite regional variations, their findings align with ours, indicating noteworthy group differences solely in the high‐frequency bands.

Our findings of frequency‐ and region‐specific abnormalities in neural network hubs may be interpreted as follows. Human cognitive functions are known to be affected by both bottom‐up and top‐down processes.^16^, ^17^, ^18^ An imbalance between these can cause hallucinations and illusions.^21^ Several studies have shown that in SZ, such imbalances are related to the background of psychiatric symptoms.^22^, ^23^, ^24^, ^25^ Recent findings show that bottom‐up and top‐down signaling use gamma and beta frequencies, respectively. Uhlhaas et al. reported that in the primate visual system, feedforward (bottom‐up) influences are carried by theta‐band and gamma‐band synchronization, whereas feedback (top‐down) influences are carried by beta‐band synchronization.^14^, ^20^ The decrease in beta‐band BC values at Fz identified here indicates reduced top‐down processes, possibly reflecting a network abnormality via the dorsolateral prefrontal cortex in patients with SZ. Interestingly, Brady et al. reported that a breakdown in connectivity between the cerebellum and the dorsolateral prefrontal cortex is associated with negative symptoms.^48^ By contrast, increased gamma‐band BC values at O1 indicate enhanced bottom‐up processes in SZ. Gamma‐band oscillations are associated with bottom‐up connectivity related to perception,^20^, ^49^ which is reportedly altered in patients with SZ.

Our finding of decreased gamma‐band BC values at Pz lacks a reasonable interpretation and requires careful discussion. Parietal lobe dysfunction has been reported in SZ^50^, ^51^ and has been implicated in causing various symptoms and characteristics of SZ, such as disorganized speech and auditory verbal hallucinations. The area around the Pz site contains the posterior cingulate cortex and precuneus, which are central brain regions in the default mode network. The default mode network reflects the functional activity of the brain at rest and is associated with functions such as emotion, memory, and attentional focus. Broadband gamma power in the default‐mode network decreases during a visual continuous performance task,^52^ therefore the alteration in BC values we observed at Pz may reflect various functional alterations that may lead to cognitive deficits in SZ.

Despite reports that antipsychotic treatment induces clinical improvements, we did not find a significant effect of antipsychotics on BC values. However, this may indicate that alterations in functional connectivity (hub status) in the high‐frequency bands reflect the intrinsic pathophysiology of SZ. Functional connectivity in high‐frequency rhythms facilitates various cognitive functions accomplished via top‐down and bottom‐up processes. Additionally, cognitive deficits are generally refractory to antipsychotic treatment, and clinical symptoms (i.e., BPRS scores) were not correlated with BC values here. We speculate that the alterations in hub status are associated with core deficits in SZ but not with treatable symptoms such as hallucinations or delusions (reflected in BPRS scores). Thus, our finding of frequency‐specific treatment‐refractory abnormal hub topologies in SZ may reflect cognitive deficits in this disorder. Our results demonstrate abnormalities in whole‐brain functional networks consistent with the disconnection hypothesis and indicate the locations of important anomalies associated with SZ.

This study has some limitations that should be considered. First, EEG data have limited spatial resolution. Additionally, our EEG measurements were performed according to typical protocols followed in real‐world clinical practice, but we used comparatively fewer electrodes. Therefore, future studies using more electrodes may provide more precise locations of anomaly centers. Second, we did not estimate the exact cognitive functions in patients with SZ. The BPRS scores used here are unsuitable for assessing cognitive function because they only measure general symptoms; the BPRS questionnaire includes only a few questions about cognitive function. Therefore, we could not elucidate a direct link between network abnormalities and cognitive functions. Third, limitations on the timing and number of EEG data measurements used here must be mentioned. No preonset data were available for the SZ patients. As such, it is unclear when these network abnormalities appear in patients with SZ. In addition, given the small number of participants in this study, it is difficult to evaluate complex interactive factors. Thus, further studies that include a larger number of participants are warranted to permit the statistical analysis of different backgrounds, and longer‐term longitudinal studies are required to interpret the present results accurately. Fourth, the notion of top‐down and bottom‐up processes requires careful discussion because BC does not reflect the directionality of neural signal propagation. It is therefore necessary to evaluate the directional functional connectivity with directed PLI to reveal the information flow for top‐down and bottom‐up at the hub of a functional network.^53^ Fifth, despite the evidence of a medication effect on functional connectivity, we did not find a significant difference in BC before and after treatment. However, this result may be due to the limitations of neuroimaging modalities and analysis methods. Additionally, the small sample sizes of the SZ groups, notably of the posttreatment group, may have admitted selection bias and influenced the result. Sixth, due to the measurement environment, the EEG data in the very high‐frequency band was not sufficient to support division of the gamma band into high‐gamma and low‐gamma bands for analysis. The effect of gamma subband distinctions on our main outcomes should therefore be investigated in further studies. Finally, regarding technical issues, applying Laplace re‐reference was appropriate here to weaken the common source problem. However, PLI analysis achieves relatively high spatial resolution without re‐reference,^15^ and re‐reference is not always necessary, notably in an extended version of PLI called wPLI.^54^ However, the actual effect of the re‐reference on the detection of the hub structure at the level of the global topology has not yet been evaluated, therefore detailed verification our main results with re‐referencing will be necessary in future studies.

## CONCLUSION

We report a significant alteration of hub structure in the neural network of drug‐naïve patients with SZ, as evaluated using resting‐state EEG rhythms in the high‐frequency bands. Despite clinical evidence demonstrating the positive effects of antipsychotic medication, the observed alterations were not ameliorated by treatment with antipsychotics. Our findings are likely related to abnormalities in the top‐down and bottom‐up processes that may contribute to cognitive deficits in SZ. These findings must be validated by longitudinal studies evaluating specific clinical symptoms and cognitive deficits associated with SZ. This study highlights the potential of a BC index based on conventional EEG data, and we demonstrate its performance in elucidating the pathophysiological bases and therapeutic mechanisms of SZ.

## AUTHOR CONTRIBUTIONS

T.I., S.N., M.K., and T.T. designed the methods. T.I., S.N., M.T., and T.T. analyzed the results, wrote the main manuscript text, and prepared all the figures. M.K. conducted the experiments, and all authors reviewed the manuscript.

## CONFLICT OF INTEREST STATEMENT

The authors declare that the research was conducted without any commercial or financial relationships that could be construed as a potential conflict of interest.

## ETHICS APPROVAL STATEMENT

The study has complied with all the relevant national regulations, institutional policies, and the tenets of the Declaration of Helsinki. It was approved by the Ethics Committee of Kanazawa University.

## PATIENT CONSENT STATEMENT

All participants who agreed to participate in the study were made aware of the research and provided written informed consent.

## CLINICAL TRIAL REGISTRATION

N/A.

## Data Availability

The datasets presented in this article are not readily available because the informed consent did not include a declaration regarding the publication of clinical data. Requests to access the datasets should be directed to Tomoaki Ishibashi (to1484@u-fukui.ac.jp) or Tetsuya Takahashi (takahash@u-fukui.ac.jp).

## References

[1] Lewis DA , Lieberman JA . Catching up on schizophrenia. Neuron. 2000;28:325–334. 10.1016/s0896-6273(00)00111-2 11144342

[2] Owen MJ , Sawa A , Mortensen PB . Schizophrenia. Lancet. 2016;388:86–97. 10.1016/S0140-6736(15)01121-6 26777917 PMC4940219

[3] Bilder RM , Lipschutz‐Broch L , Reiter G , Geisler SH , Mayerhoff DI , Lieberman JA . Intellectual deficits in first‐episode schizophrenia: evidence for progressive deterioration. Schizophr Bull. 1992;18:437–448. 10.1093/schbul/18.3.437 1411331

[4] Tandon R , Gaebel W , Barch DM , Bustillo J , Gur RE , Heckers S , et al. Definition and description of schizophrenia in the DSM‐5. Schizophr Res. 2013;150:3–10. 10.1016/J.SCHRES.2013.05.028 23800613

[5] Green MF . What are the functional consequences of neurocognitive deficits in schizophrenia? Am J Psychiatry. 1996;153:321–330. 10.1176/AJP.153.3.321 8610818

[6] Kahn RS , Keefe RSE . Schizophrenia is a cognitive illness: time for a change in focus. JAMA Psychiatry. 2013;70:1107–1112. 10.1001/JAMAPSYCHIATRY.2013.155 23925787

[7] Keefe RSE . Neurocognitive effects of antipsychotic medications in patients with chronic schizophrenia in the CATIE Trial. Arch Gen Psychiatry. 2007;64:633–647. 10.1001/ARCHPSYC.64.6.633 17548746

[8] Vita A , Barlati S , Ceraso A , Nibbio G , Ariu C , Deste G , et al. Effectiveness, core elements, and moderators of response of cognitive remediation for schizophrenia: a systematic review and meta‐analysis of randomized clinical trials. JAMA Psychiatry. 2021;78:848–858. 10.1001/JAMAPSYCHIATRY.2021.0620 33877289 PMC8058696

[9] Friston KJ . The disconnection hypothesis. Schizophr Res. 1998;30:115–125. 10.1016/S0920-9964(97)00140-0 9549774

[10] Wu XL , Yan QJ , Zhu F . Abnormal synaptic plasticity and impaired cognition in schizophrenia. World J Psychiatry. 2022;12:541–557. 10.5498/wjp.v12.i4.541 35582335 PMC9048451

[11] Li M , Chen Z , Li T . Small‐world brain networks in schizophrenia. Shanghai Arch Psychiatry. 2012;24:322–327. 10.3969/j.issn.1002-0829.2012.06.003 25324636 PMC4198898

[12] Howes OD , Kapur S . The dopamine hypothesis of schizophrenia: version III–the final common pathway: version 3. Schizophr Bull. 2009;35:549–562. 10.1093/SCHBUL/SBP006 19325164 PMC2669582

[13] Balu DT . The NMDA receptor and schizophrenia: from pathophysiology to treatment. Adv Pharmacol. 2016;76:351–382. 10.1016/BS.APHA.2016.01.006 27288082 PMC5518924

[14] Uhlhaas PJ , Haenschel C , Nikolic D , Singer W . The role of oscillations and synchrony in cortical networks and their putative relevance for the pathophysiology of schizophrenia. Schizophr Bull. 2008;34:927–943. 10.1093/SCHBUL/SBN062 18562344 PMC2632472

[15] Takahashi T , Goto T , Nobukawa S , Tanaka Y , Kikuchi M , Higashima M , et al. Abnormal functional connectivity of high‐frequency rhythms in drug‐naïve schizophrenia. Clin Neurophysiol. 2018;129:222–231. 10.1016/j.clinph.2017.11.004 29202390

[16] Kveraga K , Ghuman AS , Bar M . Top‐down predictions in the cognitive brain. Brain Cogn. 2007;65:145–168. 10.1016/J.BANDC.2007.06.007 17923222 PMC2099308

[17] Manita S , Suzuki T , Homma C , Matsumoto T , Odagawa M , Yamada K , et al. A top‐down cortical circuit for accurate sensory perception. Neuron. 2015;86:1304–1316. 10.1016/J.NEURON.2015.05.006 26004915

[18] Jo HG , Kellermann T , Baumann C , Ito J , Schulte Holthausen B , Schneider F , et al. Distinct modes of top‐down cognitive processing in the ventral visual cortex. Neuroimage. 2019;193:201–213. 10.1016/J.NEUROIMAGE.2019.02.068 30849527

[19] Wang XJ . Neurophysiological and computational principles of cortical rhythms in cognition. Physiol Rev. 2010;90:1195–1268. 10.1152/PHYSREV.00035.2008 20664082 PMC2923921

[20] Bastos AM , Vezoli J , Bosman CA , Schoffelen JM , Oostenveld R , Dowdall JR , et al. Visual areas exert feedforward and feedback influences through distinct frequency channels. Neuron. 2015;85:390–401. 10.1016/J.NEURON.2014.12.018 25556836

[21] Allen P , Larøi F , McGuire PK , Aleman A . The hallucinating brain: a review of structural and functional neuroimaging studies of hallucinations. Neurosci Biobehav Rev. 2008;32:175–191. 10.1016/j.neubiorev.2007.07.012 17884165

[22] Aleman A , Böcker KBE , Hijman R , de Haan EHF , Kahn RS . Cognitive basis of hallucinations in schizophrenia: role of top‐down information processing. Schizophr Res. 2003;64:175–185. 10.1016/S0920-9964(03)00060-4 14613682

[23] Merabet LB , Maguire D , Warde A , Alterescu K , Stickgold R , Pascual‐Leone A . Visual hallucinations during prolonged blindfolding in sighted subjects. J Neuroophthalmol. 2004;24:109–113. 10.1097/00041327-200406000-00003 15179062

[24] Dima D , Dietrich DE , Dillo W , Emrich HM . Impaired top‐down processes in schizophrenia: a DCM study of ERPs. Neuroimage. 2010;52:824–832. 10.1016/J.NEUROIMAGE.2009.12.086 20056155

[25] Linszen MMJ , Brouwer RM , Heringa SM , Sommer IE . Increased risk of psychosis in patients with hearing impairment: review and meta‐analyses. Neurosci Biobehav Rev. 2016;62:1–20. 10.1016/J.NEUBIOREV.2015.12.012 26743858

[26] Fraschini M , Demuru M , Crobe A , Marrosu F , Stam CJ , Hillebrand A . The effect of epoch length on estimated EEG functional connectivity and brain network organisation. J Neural Eng. 2016;13:036015. 10.1088/1741-2560/13/3/036015 27137952

[27] Nobukawa S , Yamanishi T , Kasakawa S , Nishimura H , Kikuchi M , Takahashi T . Classification methods based on complexity and synchronization of electroencephalography signals in Alzheimer's disease. Front Psychiatry. 2020;11:255. 10.3389/fpsyt.2020.00255 32317994 PMC7154080

[28] Stam CJ , Nolte G , Daffertshofer A . Phase lag index: assessment of functional connectivity from multi channel EEG and MEG with diminished bias from common sources. Hum Brain Mapp. 2007;28:1178–1193. 10.1002/HBM.20346 17266107 PMC6871367

[29] Freeman LC . Centrality in social networks conceptual clarification. Soc Networks. 1978;1:215–239. 10.1016/0378-8733(78)90021-7

[30] Rubinov M , Sporns O . Complex network measures of brain connectivity: uses and interpretations. Neuroimage. 2010;52:1059–1069. 10.1016/J.NEUROIMAGE.2009.10.003 19819337

[31] van Mieghem P , Magdalena SM . Phase transition in the link weight structure of networks. Phys Rev E. 2005;72:056138. 10.1103/PhysRevE.72.056138 16383719

[32] Wang H , Hernandez JM , van Mieghem P . Betweenness centrality in a weighted network. Phys Rev E. 2008;77:046105. 10.1103/PhysRevE.77.046105 18517688

[33] Stam CJ , Tewarie P , van Dellen E , van Straaten ECW , Hillebrand A , van Mieghem P . The trees and the forest: characterization of complex brain networks with minimum spanning trees. Int J Psychophysiol. 2014;92:129–138. 10.1016/J.IJPSYCHO.2014.04.001 24726900

[34] Tewarie P , van Dellen E , Hillebrand A , Stam CJ . The minimum spanning tree: an unbiased method for brain network analysis. Neuroimage. 2015;104:177–188. 10.1016/J.NEUROIMAGE.2014.10.015 25451472

[35] Benjamini Y , Hochberg Y . Controlling the false discovery rate: a practical and powerful approach to multiple testing. J R Stat Soc Ser B. 1995;57:289–300. 10.1111/J.2517-6161.1995.TB02031.X

[36] Lehmann D , Faber PL , Pascual‐Marqui RD , Milz P , Herrmann WM , Koukkou M , et al. Functionally aberrant electrophysiological cortical connectivities in first episode medication‐naïve schizophrenics from three psychiatry centers. Front Hum Neurosci. 2014;8:635. 10.3389/FNHUM.2014.00635 25191252 PMC4138932

[37] Michel CM , Koenig T . EEG microstates as a tool for studying the temporal dynamics of whole‐brain neuronal networks: a review. Neuroimage. 2018;180:577–593. 10.1016/J.NEUROIMAGE.2017.11.062 29196270

[38] Mackintosh AJ , Borgwardt S , Studerus E , Riecher‐Rössler A , de Bock R , Andreou C . EEG microstate differences in medicated vs. medication‐naïve first‐episode psychosis patients. Front Psychiatry. 2020;11:600606. 10.3389/fpsyt.2020.600606 33329154 PMC7732503

[39] Takahashi T , Cho RY , Mizuno T , Kikuchi M , Murata T , Takahashi K , et al. Antipsychotics reverse abnormal EEG complexity in drug‐naïve schizophrenia: a multiscale entropy analysis. Neuroimage. 2010;51:173–182. 10.1016/j.neuroimage.2010.02.009 20149880 PMC2849166

[40] Kambeitz J , Kambeitz‐Ilankovic L , Cabral C , Dwyer DB , Calhoun VD , van den Heuvel MP , et al. Aberrant functional whole‐brain network architecture in patients with schizophrenia: a meta‐analysis. Schizophr Bull. 2016;42(Suppl 1):S13–S21. 10.1093/SCHBUL/SBV174 27460615 PMC4960431

[41] Crossley NA , Mechelli A , Scott J , Carletti F , Fox PT , Mcguire P , et al. The hubs of the human connectome are generally implicated in the anatomy of brain disorders. Brain. 2014;137:2382–2395. 10.1093/BRAIN/AWU132 25057133 PMC4107735

[42] Klauser P , Baker ST , Cropley VL , Bousman C , Fornito A , Cocchi L , et al. White matter disruptions in schizophrenia are spatially widespread and topologically converge on brain network hubs. Schizophr Bull. 2016;43:sbw100. 10.1093/SCHBUL/SBW100 PMC5605265 27535082

[43] van den Heuvel MP , Mandl RCW , Stam CJ , Kahn RS , Hulshoff Pol HE . Aberrant frontal and temporal complex network structure in schizophrenia: a graph theoretical analysis. J Neurosci. 2010;30:15915–15926. 10.1523/JNEUROSCI.2874-10.2010 21106830 PMC6633761

[44] Cheng H , Newman S , Goñi J , Kent JS , Howell J , Bolbecker A , et al. Nodal centrality of functional network in the differentiation of schizophrenia. Schizophr Res. 2015;168:345–352. 10.1016/j.schres.2015.08.011 26299706 PMC4591247

[45] Crossley NA , Mechelli A , Ginestet C , Rubinov M , Bullmore ET , Mcguire P . Altered hub functioning and compensatory activations in the connectome: a meta‐analysis of functional neuroimaging studies in schizophrenia. Schizophr Bull. 2016;42:434–442. 10.1093/SCHBUL/SBV146 26472684 PMC4753609

[46] Krukow P , Jonak K , Karpiński R , Karakuła‐Juchnowicz H . Abnormalities in hubs location and nodes centrality predict cognitive slowing and increased performance variability in first‐episode schizophrenia patients. Sci Rep. 2019;9:9594. 10.1038/s41598-019-46111-0 31270391 PMC6610093

[47] Jonak K , Krukow P , Jonak KE , Grochowski C , Karakuła‐Juchnowicz H . Quantitative and qualitative comparison of EEG‐based neural network organization in two schizophrenia groups differing in the duration of illness and disease burden: graph analysis with application of the minimum spanning tree. Clin EEG Neurosci. 2019;50:231–241. 10.1177/1550059418807372 30322279

[48] Brady RO , Gonsalvez I , Lee I , Öngür D , Seidman LJ , Schmahmann JD , et al. Cerebellar‐prefrontal network connectivity and negative symptoms in schizophrenia. Am J Psychiatry. 2019;176:512–520. 10.1176/appi.ajp.2018.18040429 30696271 PMC6760327

[49] Gandal MJ , Edgar JC , Klook K , Siegel SJ . Gamma synchrony: towards a translational biomarker for the treatment‐resistant symptoms of schizophrenia. Neuropharmacology. 2012;62:1504–1518. 10.1016/j.neuropharm.2011.02.007 21349276 PMC3264822

[50] Torrey EF . Schizophrenia and the inferior parietal lobule. Schizophr Res. 2007;97:215–225. 10.1016/j.schres.2007.08.023 17851044

[51] Chieffi S , Ilardi CR , Iavarone A . Parietal lobe dysfunction in schizophrenia: a review. Curr Psychiatry Rev. 2018;14:71–83. 10.2174/1573400514666180703150804

[52] Li J , Kronemer SI , Herman WX , Kwon H , Ryu JH , Micek C , et al. Default mode and visual network activity in an attention task: direct measurement with intracranial EEG. Neuroimage. 2019;201:116003. 10.1016/J.NEUROIMAGE.2019.07.016 31295566 PMC8441717

[53] Stam CJ , van Straaten ECW . The organization of physiological brain networks. Clin Neurophysiol. 2012;123:1067–1087. 10.1016/J.CLINPH.2012.01.011 22356937

[54] Cohen MX . Effects of time lag and frequency matching on phase‐based connectivity. J Neurosci Methods. 2015;250:137–146. 10.1016/J.JNEUMETH.2014.09.005 25234308

